# Drug-Coated Balloon Versus Plain Old Balloon Angioplasty in Isolated Popliteal and/or Superficial Femoral Artery Disease: A Retrospective Single-Center Comparative Cohort Study

**DOI:** 10.3390/jcm15135152

**Published:** 2026-07-02

**Authors:** Volkan Tasci, Erbil Arik, Muhammed Said Besler, Ali Fuat Tekin, Mehmet Ali Durmus, Hakan Adakan, Onur Taydas, Mustafa Ozdemir, Omer Faruk Topaloglu, Mehmet Halil Öztürk

**Affiliations:** 1 Department of Radiology, Division of Interventional Radiology, Faculty of Medicine, Sakarya University, 54100 Sakarya, Turkey; md.volkantasci@gmail.com (V.T.); m.alidurmus0@gmail.com (M.A.D.); hakan.adakan1@gmail.com (H.A.); drmstfrd@gmail.com (M.O.); ofaruktopaloglu@gmail.com (O.F.T.); ozturkmh@gmail.com (M.H.Ö.); 2 Department of Radiology, Division of Interventional Radiology, Faculty of Medicine, Marmara University, 34899 Istanbul, Turkey; erbilarik@hotmail.com; 3 Department of Radiology, Division of Interventional Radiology, Faculty of Medicine, Medeniyet University, 34722 Istanbul, Turkey; msbesler@gmail.com; 4 The Russell H. Morgan Department of Radiology and Radiological Sciences, School of Medicine, Johns Hopkins University, Baltimore, MD 21205, USA; aftrad333@gmail.com

**Keywords:** popliteal artery, superficial femoral artery, drug-coated balloon, paclitaxel, endovascular therapy, peripheral artery disease, ankle–brachial index, Rutherford classification, real-world

## Abstract

**Background/Objectives**: Drug-coated balloons (DCBs) deliver paclitaxel to the vessel wall and leave nothing behind, which makes them mechanistically appealing here, yet head-to-head data confined to the isolated popliteal/SFA segment are still scarce. We directly compared 12-month hemodynamic, symptomatic, and patency loss outcomes between DCB and plain old balloon angioplasty (POBA) in this anatomical setting. **Methods**: We retrospectively reviewed 401 consecutive endovascular procedures performed at a single center between January 2021 and December 2024 for isolated popliteal and/or SFA disease, comprising 179 DCB and 222 POBA cases. 12-month endpoints of composite clinical success, asymptomatic recovery, and composite patency loss were analyzed. The composite patency loss endpoint was further fitted to a multivariable logistic regression with baseline ABI, baseline Rutherford category, lesion length, and total occlusion as covariates. The composite patency loss endpoint was further fitted to a multivariable logistic regression with baseline ABI, baseline Rutherford category, lesion length, and total occlusion as covariates, designated as the principal effect estimate. Kaplan–Meier cumulative incidence plots are presented descriptively only. **Results**: The study population comprised 401 patients (mean age 68.4 ± 10.6 years; 316 male [78.8%]), with 179 in the DCB arm (mean age 65.3 ± 10.5 years; 80.4% male) and 222 in the POBA arm (mean age 71.0 ± 9.8 years; 77.4% male). DCB-treated lesions started out more advanced: longer (94.5 ± 48.2 vs. 82.7 ± 43.3 mm; *p* = 0.010), more often totally occluded (39.7% vs. 19.4%; *p* < 0.001), and weighted toward TASC II C/D (*p* < 0.001). Mean ABI improved by +0.27 in both arms, with no detectable between-arm difference (*p* = 0.860; within-arm *p* < 0.001 in each). Asymptomatic recovery at 12 months was more common after DCB (62.0% vs. 51.4%; *p* = 0.033; OR 1.55, 95% CI 1.04–2.31), and composite patency loss was roughly halved (6.7% vs. 12.6%; *p* = 0.050; OR 0.50, 95% CI 0.25–1.01). Documented TLR (4.5% vs. 7.2%; *p* = 0.251) and composite clinical success (86.6% vs. 82.4%; *p* = 0.255) did not reach significance. **Conclusions**: Across 401 real-world procedures in isolated popliteal and/or SFA disease, mean ABI gain was identical between arms, yet DCB delivered measurably more complete symptomatic recovery and a near-significant halving of composite patency loss at 12 months, with both signals robust to multivariable adjustment. In this real-world setting, DCB was associated with more complete symptomatic recovery and a numerically lower composite patency loss rate; these findings are hypothesis-generating and require confirmation in adequately powered randomized trials.

## 1. Introduction

Atherosclerotic disease of the femoropopliteal axis is the most common anatomical pattern in symptomatic peripheral artery disease (PAD) and accounts for much of the global disability and amputation burden attributable to PAD [1,2]. The two segments differ mechanically. The SFA is loaded by torsional and longitudinal forces from hip and knee motion; the popliteal artery is bent, compressed, and kinked across the knee with every step. This cyclical strain underlies the reputation of the popliteal artery as a “no-stent zone”: self-expanding stents fracture, restenosis, and thrombosis more readily here than almost anywhere else in the peripheral circulation [3,4,5,6].

Plain old balloon angioplasty (POBA) served as the default endovascular treatment in this territory for decades, but elastic recoil, dissection, and the lack of any antiproliferative effect leave 40–60% of treated segments restenosed within the first year [7,8]. Drug-coated balloons (DCBs) deliver paclitaxel to the vessel wall during a brief inflation and were developed to suppress neointimal hyperplasia without leaving a permanent implant. In the popliteal segment, where stent-related complications are most punishing, this “leave-nothing-behind” approach fits both the biological and anatomical demands of the popliteal segment [9,10].

Randomized and registry evidence increasingly favors DCB over POBA in the femoropopliteal territory. The IN.PACT SFA trial demonstrated a durable patency advantage for paclitaxel-coated balloons in SFA disease [7], and the long-term IN.PACT Global data carried this signal into the popliteal segment [11]. K-POP, in 100 popliteal-artery patients treated with the IN.PACT Admiral DCB, reported a 12-month clinical primary patency of 76.0% and a TLR-free rate of 87.2%, with female sex and lesion length emerging as independent predictors of patency loss [12]. Single-center series from Europe [13,14], China [15], and Korea [16] echo this picture but rest on heterogeneous endpoint definitions and tend to report continuous patency rather than patient-level binary outcomes.

Real-world head-to-head comparisons confined to the isolated popliteal/SFA territory, with patient-level binary endpoints and appropriate multivariable adjustment, are still hard to find. The present study was designed with this void in mind: we compared 12-month hemodynamic, symptomatic, and patency loss outcomes between DCB and POBA in a consecutive single-center cohort restricted to isolated popliteal and/or SFA disease, with unadjusted and adjusted treatment-effect estimates for the primary endpoint.

## 2. Materials and Methods

### 2.1. Study Design and Population

This retrospective, single-center comparative cohort study was conducted using prospectively maintained endovascular procedure registries. All consecutive adult patients who underwent endovascular treatment for isolated popliteal and/or SFA atherosclerotic disease between 1 January 2021 and 31 December 2024 were screened. Two treatment arms were prespecified: a drug-coated balloon (DCB) arm (*n* = 179) and a plain old balloon angioplasty (POBA) arm (*n* = 222), the latter comprising all patients in whom plain balloon dilatation without paclitaxel coating was used as the definitive therapeutic modality. The study was conducted in accordance with the Declaration of Helsinki and approved by the Health Sciences Scientific Research Ethics Committee of Sakarya University (protocol code: E-43012747-050.04-489676-394; date of approval: 8 July 2025). Written informed consent was waived owing to the retrospective design and the use of fully de-identified data.

### 2.2. Inclusion and Exclusion Criteria

Patients were eligible for inclusion if they met all of the following criteria:

- Age ≥ 18 years;
- Symptomatic PAD with Rutherford category 2–5;
- Angiographically confirmed atherosclerotic stenosis (>50%) of the popliteal artery (segments P1–P3) and/or the SFA, with or without contiguous infrapopliteal involvement;
- Treatment with a single endovascular balloon strategy (DCB or POBA) without primary stent implantation;
- Availability of complete clinical and ABI follow-up at both 6 and 12 months together with documented Rutherford classification at baseline and at 12 months.

Exclusion criteria were as follows:

- Multilevel disease requiring concomitant proximal endovascular or surgical revascularization beyond the index target lesion;
- Previous popliteal artery stenting;
- Hybrid open–endovascular procedures;
- Acute limb ischemia;
- Rutherford category 6 (extensive tissue loss);
- Congestive heart failure with left ventricular ejection fraction < 40%;
- Life expectancy < 12 months due to comorbidity;
- Incomplete clinical, hemodynamic, or imaging follow-up;
- Bailout/provisional stenting required during the index procedure.

Patients in whom adjunctive directional or rotational atherectomy was performed (*n* = 5; 4 in the DCB arm and 1 in the POBA arm) were retained in their respective treatment arms but, given the small subgroup size, were not analyzed separately.

### 2.3. Endovascular Procedure

All procedures were performed by five experienced interventional radiologists (with 4, 5, 9, 13 and 25 years of experience) in a hybrid catheterization laboratory under local anesthesia with conscious sedation. Vascular access was obtained percutaneously through the common femoral artery using either ipsilateral antegrade puncture or contralateral cross-over according to lesion location and inflow anatomy. A 6F or 7F introducer sheath was placed, and intravenous unfractionated heparin was administered at 70–100 IU/kg to maintain an activated clotting time > 250 s. Diagnostic angiography of the entire arterial tree from the ipsilateral superficial femoral artery to the pedal vessels was obtained at the start of every procedure. After successful crossing of the target lesion with a 0.018 or 0.035 guidewire, pre-dilatation with an appropriately sized, semi-compliant plain balloon was performed in all patients to assess vessel response and rule out flow-limiting dissection.

In the DCB arm, definitive treatment was performed with a DCB inflated for at least 3 min at nominal pressure, with the balloon length and diameter selected to overlap the entire lesion plus 1 cm of healthy proximal and distal reference vessel. In the POBA arm, definitive treatment consisted of standard plain balloon dilatation with prolonged inflation (≥2 min) at the operator’s discretion. Provisional bailout stenting was used only in cases of flow-limiting dissection or residual stenosis > 50%; these patients were excluded from the principal analysis. Adjunctive atherectomy was used in 5 patients overall (1.2%); these patients were retained in their respective treatment arms. Procedural success was defined as residual stenosis < 30% on completion angiography without flow-limiting dissection or distal embolization. Post-procedurally, all patients received acetylsalicylic acid 100 mg indefinitely and clopidogrel 75 mg daily for a minimum of six months [17,18].

### 2.4. Follow-Up Protocol

Patients were followed up clinically at 1, 3, 6, 9, and 12 months. Resting ABI was measured at the time of discharge and at the 6-month and 12-month visits. Rutherford category was reassessed at every visit, with the post-treatment Rutherford classification reported at the 12-month visit. Imaging—either duplex ultrasonography or computed tomography angiography—was obtained on a clinical-trigger basis: in patients with persistent or recurrent symptoms, with a decrease in ABI ≥ 0.15 from the post-procedural value, or with a worsening Rutherford category. Routine surveillance imaging was not protocol-mandated.

### 2.5. Endpoints and Definitions

Three prespecified 12-month binary endpoints were analyzed. (i) Composite clinical success was defined as the simultaneous achievement of an absolute ABI increase ≥ 0.10 from baseline and an improvement of ≥1 Rutherford category. (ii) Asymptomatic recovery was defined as a 12-month Rutherford category of 0. Rutherford category 0 was selected as the threshold for asymptomatic recovery because it represents complete symptomatic resolution—the most clinically meaningful individual-level outcome in a claudicant population. Composite patency loss was defined as the occurrence of any of the following: hemodynamic deterioration (ABI decrease ≥ 0.15 between the 6-month and 12-month measurements or absolute ABI gain from baseline < 0.10); worsening of the Rutherford category between baseline and 12 months; or a documented clinically driven target lesion revascularization (TLR), defined as repeat endovascular or surgical revascularization of the index lesion driven by recurrent symptoms together with an ABI decrease ≥ 0.15. All three components are directionally defined as treatment failure or clinical deterioration, distinguishing CPL from other reported endpoints—composite clinical success and asymptomatic recovery—which capture therapeutic benefit. Although CPL shares measurement instruments (ABI, Rutherford category, TLR record) with these endpoints, the thresholds and directionality are non-overlapping. The hemodynamic deterioration and Rutherford worsening components were adjudicated on the basis of scheduled-visit ABI measurement and clinical examination alone, independently of imaging, and were therefore assessed uniformly in all patients who completed follow-up. The TLR component additionally required procedural confirmation, which presupposes that clinically triggered imaging had been obtained; silent restenosis that produced neither ABI decline nor symptomatic worsening sufficient to prompt imaging would not have been captured under this component. The inclusion of absolute ABI gain < 0.10 as a hemodynamic failure criterion was intended to capture patients in whom the procedure produced no clinically meaningful hemodynamic benefit, regardless of whether a prior gain had subsequently been lost; we acknowledge that this component identifies non-improvers as well as patients with true patency deterioration, and that a restricted composite of Rutherford worsening and TLR alone would more narrowly approximate conventional patency loss definitions. Continuous secondary endpoints comprised mean ABI at baseline, 6 months, and 12 months; ΔABI from baseline to 12 months; and median Rutherford classification at baseline and at 12 months. Safety endpoints comprised death, major amputation, surgical revascularization of the index lesion, and major procedural complications.

### 2.6. Statistical Analysis

Continuous variables were tested for normality with the Shapiro–Wilk test and reported as mean ± standard deviation (SD) when symmetrically distributed and as median (interquartile range, IQR) otherwise. Categorical variables were reported as counts and percentages. Between-group comparisons used the independent-samples *t*-test or the Mann–Whitney U-test for continuous variables, and the chi-square test (or Fisher’s exact test where any expected cell count was <5) for categorical variables. Within-group comparisons of ABI between baseline and follow-up time points used the paired-samples *t*-test, and within-group comparisons of Rutherford classification used the Wilcoxon signed-rank test. The longitudinal effect of treatment on ABI was evaluated with repeated-measures analysis of variance (RM-ANOVA), with treatment group and time as factors. Kaplan–Meier cumulative incidence plots for clinically driven TLR-free status and freedom from composite patency loss are presented descriptively to visualize the visit-anchored event-accrual pattern over the 12-month follow-up. Because events were interval-censored at the scheduled 6- and 12-month visits and interval imaging was clinically triggered rather than protocol-mandated, no hazard ratios, confidence intervals on the survival scale, or log-rank tests were derived from these plots, and a continuous-time Cox proportional hazards model was not undertaken. Twelve-month outcomes are therefore reported as binary endpoints with unadjusted odds ratios from 2 × 2 contingency tables and a multivariable logistic regression as the principal effect estimate. The primary effect of treatment on each binary endpoint was additionally quantified with the unadjusted odds ratio (OR) and 95% CI from a 2 × 2 contingency table. To account for between-group imbalances in baseline clinical severity and lesion morphology, a multivariable logistic regression model was fitted with the composite patency loss endpoint as the dependent variable and the following covariates: treatment arm (DCB vs. POBA), baseline ABI (continuous, per 0.1-unit increment), baseline Rutherford category ≥ 4 (binary), lesion length (continuous, per 10 mm increment), and total occlusion (binary). Severe calcification, run-off vessels ≤ 1, and wiring approach were assessed as additional covariates in a sensitivity analysis. Two additional prespecified sensitivity logistic regressions were also fitted to address the between-arm imbalance in chronic kidney disease and age: an expanded model incorporating CKD (binary) and age (continuous, per 5-year increment) alongside the original covariates, and a parsimonious model retaining only treatment arm, age, CKD, and baseline Rutherford ≥ 4. The parsimonious model was included to ensure an adequate events-per-variable ratio, given approximately 40 composite-patency loss events. Two-sided *p*-values < 0.05 were considered statistically significant. Analyses were performed using SPSS Statistics version 26 (IBM Corp., Armonk, NY, USA), R version 4.3 with the survival and lifelines packages, and Python 3.11 with statsmodels.

## 3. Results

### 3.1. Baseline Demographics and Comorbidities

**Figure 1:**
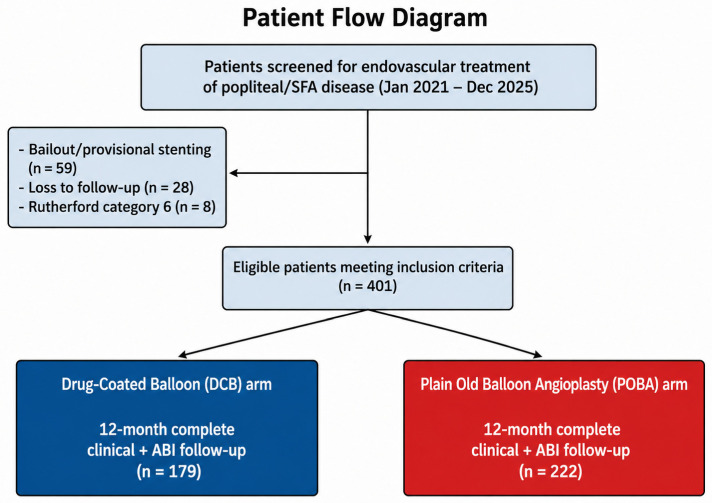
CONSORT-style patient flow diagram showing screening, sequential exclusion by reason (bailout/provisional stenting and loss to 6- or 12-month clinical or ABI follow-up, both with arm-stratified counts; Rutherford category 6), eligibility, allocation, and analyzed populations. Of patients excluded for bailout/provisional stenting, 25 were DCB-intended and 34 were POBA-intended; of patients excluded for incomplete 6- or 12-month follow-up, 12 were DCB-intended and 16 were POBA-intended. Abbreviations: ABI, ankle–brachial index; DCB, drug-coated balloon; POBA, plain old balloon angioplasty; SFA, superficial femoral artery.

Of the 401 patients who met inclusion criteria during the study period, 179 received DCB and 222 received POBA. The CONSORT-style flow of screening, exclusion (bailout/provisional stenting *n* = 59, with 25 DCB-intended and 34 POBA-intended; loss to follow-up at 6 or 12 months *n* = 28, with 12 DCB-intended and 16 POBA-intended; Rutherford category 6 *n* = 8), eligibility, allocation, and analysis is presented in Figure 1. Arm-stratified loss-to-follow-up rates were 12/191 (6.3%) in the DCB-intended arm and 16/238 (6.7%) in the POBA-intended arm and did not differ between treatment arms (chi-square *p* = 0.853). Baseline demographic and comorbidity characteristics appear in Table 1. The POBA cohort was older than the DCB cohort (mean age 71.0 ± 9.8 vs. 65.3 ± 10.5 years; *p* < 0.001) and carried more chronic kidney disease (CKD; 28.8% vs. 17.8%; *p* = 0.009). Male sex was similarly common in both arms (POBA 77.4% vs. DCB 80.4%; *p* = 0.462). Hypertension, heart failure, diabetes mellitus, coronary artery disease, and active smoking did not differ between groups (all *p* > 0.05). The age and CKD imbalance reflects a real-world allocation pattern in which sicker, older patients tended to receive plain balloon angioplasty.

### 3.2. Lesion and Procedural Characteristics

Lesion morphology and procedural data are tabulated in Table 2. By several measures, the DCB arm received the harder anatomical targets. Mean lesion length was longer (94.5 ± 48.2 mm vs. 82.7 ± 43.3 mm; *p* = 0.010), total occlusion was nearly twice as prevalent (39.7% vs. 19.4%; *p* < 0.001), and the TASC II distribution was shifted toward the more advanced classes (TASC II C 30.2% vs. 12.6%; TASC II D 11.7% vs. 6.8%; overall *p* < 0.001). Severe calcification (DCB 22.3% vs. POBA 24.8%; *p* = 0.652), the proportion of patients with one or no patent infrapopliteal run-off vessels (38.5% vs. 34.7%; *p* = 0.487), and the wiring approach (intraluminal 83.8% vs. 87.8%; *p* = 0.310) were comparable. Among patients with popliteal involvement, the P2 segment was most often affected (DCB 77.4% vs. POBA 75.0%), with P1 and P3 trailing and no between-arm differences (all *p* > 0.5). Combined SFA + popliteal disease was more common in the DCB arm (26.8% vs. 16.7%; *p* = 0.018), while contiguous infrapopliteal angioplasty was used more often in the POBA arm (62.6% vs. 52.0%; *p* = 0.038). Reference vessel diameters and balloon nominal diameters were comparable between arms. Inflation time was significantly longer in the DCB arm (3.1 ± 0.4 min vs. 2.4 ± 0.5 min; *p* < 0.001), reflecting the protocol-mandated minimum 3 min paclitaxel-balloon inflation. Procedural success was 99.4% in the DCB arm and 99.1% in the POBA arm (*p* = 1.000). Adjunctive directional or rotational atherectomy was used in 5 patients (4 DCB, 1 POBA; 1.2% of the overall cohort); these patients were retained in their treatment arms, but the small subgroup precluded a formal sub-analysis. Provisional bailout stenting cases were excluded from the principal analysis.

### 3.3. Hemodynamic Outcomes (ABI)

**Figure 2:**
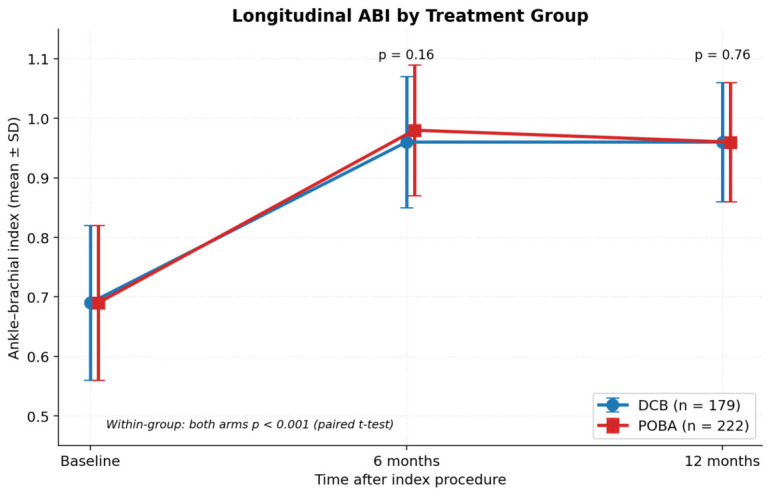
Mean ankle–brachial index (±SD) by treatment group at baseline, 6 months, and 12 months. Both arms achieved significant within-group improvement (paired *t*-test *p* < 0.001); between-group comparisons at each time point were not statistically significant. DCB, drug-coated balloon; POBA, plain old balloon angioplasty; SD, standard deviation.

Hemodynamic and clinical outcomes are tabulated in Table 3 and shown in Figure 2. Baseline ABI was identical between the two arms (mean 0.69 ± 0.13 in both; *p* = 0.892). Each arm produced an unambiguous within-group ABI gain at 6 and 12 months (paired *t*-test *p* < 0.001 for both arms at both time points). Between-arm ABI did not differ at 6 months (DCB 0.96 ± 0.11 vs. POBA 0.98 ± 0.11; *p* = 0.162) or at 12 months (DCB 0.96 ± 0.10 vs. POBA 0.96 ± 0.10; *p* = 0.757). The mean baseline-to-12-month change (ΔABI) was the same in both arms (+0.27 ± 0.08; *p* = 0.860).

### 3.4. Symptomatic Outcomes (Rutherford Classification)

**Figure 3:**
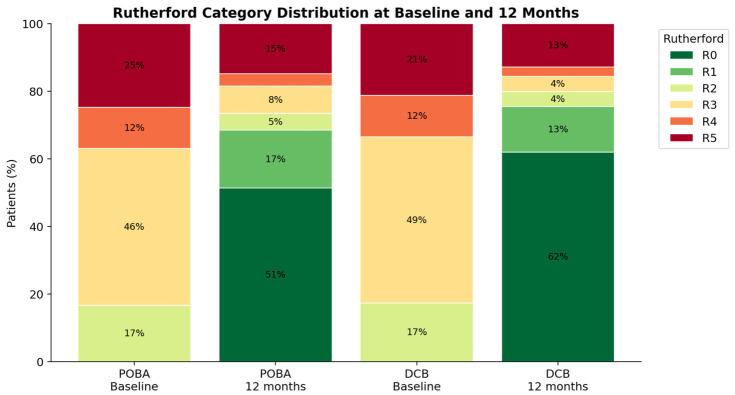
Stacked-bar distribution of Rutherford categories at baseline and 12 months for each treatment arm (Rutherford 0–5; Rutherford 6 was an exclusion criterion and is not present). Mann–Whitney U-test for between-group post-treatment Rutherford distribution: *p* = 0.048. DCB, drug-coated balloon; POBA, plain old balloon angioplasty.

The Rutherford distributions at baseline and at 12 months are shown in Figure 3 Both arms started at a median Rutherford category of 3 (IQR 3–4; *p* = 0.493). At 12 months, both medians had collapsed to 0, but the full Rutherford distribution at 12 months still favored DCB (Mann–Whitney U *p* = 0.048). Asymptomatic recovery (Rutherford 0) was reached more often in the DCB arm (62.0% vs. 51.4%; *p* = 0.033; Table 3 and Table 4). At least one Rutherford-category improvement was reached at similar rates in the two arms (DCB 87.2% vs. POBA 84.2%; *p* = 0.409).

### 3.5. Composite Patency Loss and Documented TLR

**Figure 4:**
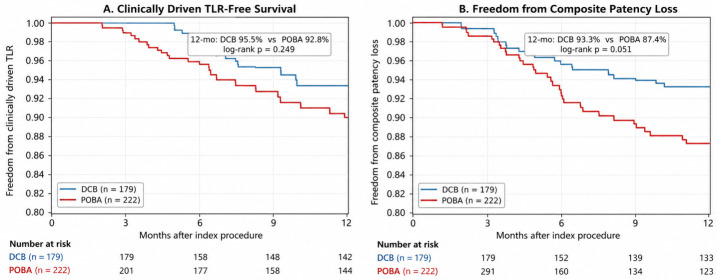
Kaplan–Meier cumulative incidence plots at the scheduled 6- and 12-month visits, presented descriptively. Panel (**A**): clinically driven TLR-free status; 12-month proportions DCB 95.5% vs. POBA 92.8%. Panel (**B**): freedom from composite patency loss (hemodynamic deterioration, defined as ABI decline ≥ 0.15 between the 6- and 12-month visits or absolute ABI gain from baseline < 0.10; Rutherford worsening; or documented clinically driven TLR); 12-month proportions DCB 93.3% vs. POBA 87.4%. Because events were interval-censored at scheduled visits and interval imaging was clinically triggered rather than protocol-mandated, hazard ratios and log-rank tests are not reported from these plots; the principal between-group inferential comparisons are reported as unadjusted odds ratios and a multivariable logistic regression (Table 4 and Section 3.6). Abbreviations: ABI, ankle–brachial index; DCB, drug-coated balloon; POBA, plain old balloon angioplasty; TLR, target lesion revascularization.

Twelve-month binary outcomes are summarized in Table 4. Kaplan–Meier cumulative incidence plots for clinically driven TLR-free status and freedom from composite patency loss are shown descriptively in Figure 4; the principal between-group inferential comparisons are reported in Table 4 and Section 3.6. Documented clinically driven TLR occurred in 8 of 179 DCB patients (4.5%) and 16 of 222 POBA patients (7.2%; chi-square *p* = 0.251; OR 0.60, 95% CI 0.25–1.44). Composite patency loss, defined to include hemodynamic deterioration, Rutherford worsening, or a documented TLR, occurred in 12 of 179 DCB patients (6.7%) versus 28 of 222 POBA patients (12.6%) (chi-square *p* = 0.050; OR 0.50, 95% CI 0.25–1.01). Asymptomatic recovery at 12 months (Rutherford 0) was reached more often in the DCB arm (62.0% vs. 51.4%; OR 1.55, 95% CI 1.04–2.31; *p* = 0.033). Composite clinical success, the combination of an ABI gain ≥ 0.10 with at least one Rutherford-category improvement, favored DCB without reaching significance (86.6% vs. 82.4%; *p* = 0.255).

### 3.6. Multivariable Logistic Regression Analysis

To handle the between-group imbalances in age, chronic kidney disease, baseline clinical severity, and lesion morphology, we fitted a multivariable logistic regression with composite patency loss as the outcome and treatment arm, baseline ABI (per 0.1 unit), baseline Rutherford ≥ 4, lesion length (per 10 mm), and total occlusion as covariates (Table 5). After adjustment, DCB carried a 49% lower odds of patency loss than POBA (adjusted OR 0.51, 95% CI 0.24–1.05; *p* = 0.065), matching the unadjusted estimate in both direction and magnitude (OR 0.50). Baseline ABI (per 0.1-unit increment, OR 1.94, 95% CI 1.29–2.93; *p* = 0.002) and baseline Rutherford ≥ 4 (OR 3.28, 95% CI 1.24–8.62; *p* = 0.016) were independent predictors of patency loss. Lesion length (OR 1.01 per 10 mm, *p* = 0.757) and total occlusion (OR 1.10, *p* = 0.818) were not independently associated with the outcome in this cohort, even though both differed between treatment arms (Table 2). A sensitivity model that additionally adjusted for severe calcification and run-off vessels ≤ 1 returned a similar adjusted OR for DCB (0.50, 95% CI 0.24–1.04; *p* = 0.065). The DCB advantage therefore persisted after accounting for the harder lesion profile in the DCB arm, which argues against the apparent benefit being a baseline-population artifact. Two additional prespecified sensitivity models addressed the between-arm imbalance in chronic kidney disease and age. In the expanded model incorporating CKD and age alongside the original covariates, the adjusted DCB-versus-POBA odds ratio for composite patency loss was 0.52 (95% CI 0.24–1.10; *p* = 0.086). In the parsimonious model (treatment arm, age, CKD, baseline Rutherford ≥ 4), the adjusted DCB odds ratio was 0.48 (95% CI 0.23–1.00; *p* = 0.051). Both sensitivity estimates were consistent in direction and magnitude with the primary adjusted model (OR 0.51), confirming that the DCB effect on composite patency loss is not explained by baseline imbalances in age or CKD prevalence between treatment arms. The expanded model carried an events-per-variable ratio of approximately 5.7, below the conventional threshold of 10; the parsimonious model is therefore reported as the more statistically stable confirmation of the same finding.

### 3.7. Safety

Complications were identified by a single investigator through retrospective review of operative notes and clinical records; formal extractor blinding was not applied, and events were not adjudicated against the Society of Interventional Radiology/Cardiovascular and Interventional Radiological Society of Europe (SIR/CIRSE) adverse event classification. Across the 12-month follow-up, there were no procedure-related deaths within 30 days, no major amputations, and no surgical bypasses of the index lesion in either arm. In the DCB cohort, the recorded complications were flow-limiting dissection in 7 patients (3.9%), acute target-vessel thrombosis in 7 (3.9%), distal embolization in 4 (2.2%), and rescue catheter-directed thrombolysis with tissue plasminogen activator in 6 (3.4%). The POBA cohort recorded distal embolization in 2 patients (0.9%); the prespecified inclusion criteria removed POBA-arm flow-limiting dissections requiring provisional bailout stenting from the principal analysis, and no other major complications were adjudicated against a uniform severity scale. Every event in both arms was salvaged either conservatively or endovascularly, with antegrade flow restored.

## 4. Discussion

The 401-patient dataset tells a fairly consistent story across four dimensions. Mean ABI rose by roughly +0.27 in each arm at 12 months, with no detectable between-arm difference but unambiguous within-arm gain (paired *t*-test *p* < 0.001 in each arm). DCB more often achieved full symptomatic recovery: 62.0% reached Rutherford 0 versus 51.4% (*p* = 0.033), and the composite 12-month patency loss rate was roughly halved, falling from 12.6% in the POBA arm to 6.7% in the DCB arm (*p* = 0.050; OR 0.50, 95% CI 0.25–1.01). The DCB cohort had the harder lesions. Lesions were longer (94.5 vs. 82.7 mm; *p* = 0.010), more often occluded (39.7% vs. 19.4%; *p* < 0.001), and weighted toward TASC II C and D (*p* < 0.001), reflecting operator preference for paclitaxel coverage in anatomically more demanding targets. After adjustment for baseline ABI, Rutherford ≥ 4, lesion length, and total occlusion, the DCB effect on patency loss held its magnitude (adjusted OR 0.51, 95% CI 0.24–1.05; *p* = 0.065). Neither the comorbidity imbalance (POBA was older with more CKD) nor the lesion-severity imbalance (longer, more occlusive lesions in DCB) accounts for the clinical advantage. Treatment allocation in our cohort was not randomized and followed a lesion-driven, operator-guided decision framework. DCB was preferentially selected for longer, more complex lesions—a pattern reflected in the significantly greater lesion length, total occlusion rate, and TASC II C/D distribution in the DCB arm—while POBA was more often chosen for shorter, less morphologically demanding lesions and for older or frailer patients in whom procedural duration and device cost were additional considerations. No formal protocol change governing device selection was introduced during the study window; however, we cannot exclude the possibility that gradual operator experience with DCB technology over the 2021–2024 period introduced a temporal drift in allocation patterns, which represents a source of residual confounding not fully captured by the multivariable model.

The comorbidity imbalance between arms—specifically the higher prevalence of CKD (28.8% vs. 17.8%; *p* = 0.009) and older age (71.0 vs. 65.3 years; *p* < 0.001) in the POBA cohort—deserves closer examination. CKD is independently associated with accelerated medial calcification, impaired endothelial repair, and a pro-inflammatory vascular milieu that amplifies neointimal hyperplasia after balloon injury. In the PAD literature, CKD has consistently emerged as an independent predictor of worse limb outcomes and higher reintervention rates after endovascular therapy, irrespective of treatment modality. In our cohort, this imbalance reflects a real-world allocation pattern: operators preferentially selected POBA for older, frailer patients with renal impairment, in whom contrast load, procedural duration, and device cost weighed against the incremental use of paclitaxel-coated technology. Critically, this allocation pattern biases against POBA—the arm with more CKD and older patients is also the arm expected to fare worse on biological grounds alone. Critically, this allocation pattern biases against POBA—the arm with more CKD and older patients is also the arm expected to fare worse on biological grounds alone. The prespecified sensitivity analyses incorporating age and CKD into the multivariable model (Section 3.6) confirm this prediction empirically: the adjusted DCB odds ratio for composite patency loss was preserved in both direction and magnitude relative to the primary model (expanded model OR 0.52; parsimonious model OR 0.48; primary OR 0.51), demonstrating that the observed DCB advantage is not an artifact of baseline imbalance in age or CKD prevalence.

Our event rates and effect sizes broadly match those in the published DCB literature, though with patient-level binary outcomes that most registry reports do not provide. K-POP [12] reported a 12-month clinical primary patency of 76.0% and a TLR-free rate of 87.2% with the IN.PACT Admiral DCB across 100 popliteal patients (mean lesion length 93.7 ± 53.7 mm; total occlusions 45.0%; TASC II C/D 32.0%); those numbers line up closely with our DCB arm (94.5 ± 48.2 mm, 39.7%, and 41.9%), suggesting direct cross-cohort comparability. In IN.PACT SFA [7], the 12-month TLR rates were 2.4% for DCB and 20.6% for POBA, and Wu and colleagues reported 72.6% 12-month primary patency in DCB-treated isolated popliteal chronic total occlusions [15]. The pooled meta-analyses by Caradu and by Giacoppo each estimate roughly a 50% reduction in restenosis odds with DCB, a magnitude that closely matches the unadjusted (OR 0.50) and adjusted (OR 0.51) odds ratios for composite patency loss in our cohort.

The notably low absolute TLR rates in our cohort warrant some explanation. Our 12-month documented TLR was 4.5% in the DCB arm and 7.2% in the POBA arm, well below the 12.8% TLR in K-POP [12], the 20.6% POBA TLR in IN.PACT SFA [7], the 14–18% pooled POBA TLR in the Caradu and Giacoppo meta-analyses [19,20], and the 9–12% DCB TLR in IN.PACT Global popliteal [11] and DAART [13]. Three local conditions help explain the gap. Surveillance imaging in our practice is clinically triggered rather than protocol-mandated at 6 and 12 months, so silent angiographic restenosis that would have been adjudicated as binary events in trial settings did not surface as TLRs here. Inclusion also required complete clinical and ABI follow-up at both visits, which selects for survivors with stable disease. And our re-intervention threshold sits on the conservative end: a patient with mild claudication recurrence and a still-acceptable ABI is more often kept on medical therapy than booked for repeat angioplasty. The composite patency loss endpoint, which incorporates hemodynamic deterioration and Rutherford worsening, captures the underlying restenosis signal more sensitively than documented TLR alone, and our composite OR of 0.50 falls within the range of meta-analytic estimates reported by Caradu and Giacoppo [19,20].

Outcomes were captured at fixed scheduled visits (baseline, 6 months, and 12 months) and on a clinical-trigger imaging basis, not as continuous time-to-event tracking. The Kaplan–Meier plots in Figure 2 are therefore presented as descriptive visit-anchored cumulative incidence visualizations rather than as inferential survival analyses. Because events were interval-censored at scheduled visits and interval imaging was clinically triggered rather than protocol-mandated, we did not derive hazard ratios, fit a proportional-hazards Cox model, or report log-rank tests; the unadjusted odds ratios and the multivariable logistic regression are the principal effect estimates [21,22].

Mechanistically, paclitaxel inhibits microtubule disassembly in vascular smooth muscle cells and suppresses the neointimal hyperplasia that drives early restenosis after balloon injury [9,10]. The biomechanical environment of the popliteal artery, with flexion-induced compression, kinking, and longitudinal strain across the knee [21], both amplifies the restenosis stimulus after POBA and punishes any permanent implant. Rastan and colleagues, in a randomized trial of stent versus balloon in popliteal disease, found only a modest benefit of stenting in this segment [3], which reinforces the rationale for a “leave-nothing-behind” strategy that combines mechanical lumen gain with antiproliferative biology. The DAART trial reported 82% versus 65% 12-month patency for atherectomy plus DCB versus DCB alone in popliteal disease [13]. Atherectomy was used as an adjunct in only 5 patients in our series and could not be analyzed as a separate stratum; this is acknowledged as a limitation when interpreting our data on vessel-preparation strategies.

Clinically, the most actionable takeaway is that ABI gain alone does not separate DCB from POBA in patients with claudication or chronic limb-threatening ischemia; what DCB delivers, in our experience, is a higher likelihood that the patient ends up symptom-free. The roughly halved odds of composite patency loss persist after adjustment, so the antiproliferative effect is unlikely to be a confounding artifact of baseline severity. Safety in our cohort was also unremarkable: no procedure-related deaths, no major amputations, and a manageable rate of dissection, distal embolization, and acute thrombosis (each at or below 4% in the DCB arm). On balance, the safety profile and clinical outcomes support DCB as a routine first choice in the popliteal “no-stent zone.”

This study has limitations that should be stated plainly. The work is retrospective, single-center, and non-randomized; even after adjustment for the most important measurable confounders, residual unmeasured factors (operator preference, device-generation effects, drift in adjunctive pharmacotherapy over the study window) cannot be fully ruled out. Formal procedural technical success criteria, including residual stenosis quantification on quantitative vascular angiography and dissection grading per the National Heart, Lung, and Blood Institute classification, were not consistently captured in our procedural records, although procedural success rates near 99% in both arms were inferable from completion angiography findings. Intravascular ultrasound was not part of the routine workflow and was not used to confirm optimal balloon sizing or residual luminal area at the lesion level. Post-treatment Rutherford classification was recorded as a single 12-month value rather than at every visit, which precluded analysis of the Rutherford trajectory over time. Surveillance imaging was clinically triggered rather than protocol-mandated, which almost certainly underestimates asymptomatic angiographic restenosis in both arms and precludes estimation of true imaging-defined primary patency. As a consequence, the reported CPL rates and TLR frequencies should be interpreted as lower bounds on true restenosis burden rather than as conventional patency endpoints; direct numerical comparisons with trials employing mandatory duplex surveillance—such as IN.PACT SFA or DAART—should account for this systematic difference in ascertainment. This adjudication structure means that ascertainment bias primarily affects the TLR component of CPL; the hemodynamic and symptomatic components, which together accounted for the majority of CPL events in both arms, were captured through scheduled non-invasive assessment and are unlikely to have been differentially missed between treatment groups. The ABI gain < 0.10 component of the CPL endpoint captures both non-improvers and patients with true hemodynamic deterioration, which may broaden the composite beyond a conventional patency loss definition. Component-level event counts were not available as discrete registry variables, precluding a restricted sensitivity composite analysis limited to Rutherford worsening and TLR. Complications were extracted from operative notes and were not adjudicated against the Society of Interventional Radiology/Cardiovascular and Interventional Radiological Society of Europe (SIR/CIRSE) classification. The 12-month window is also short for a paclitaxel-DCB analysis; longer-horizon (3- and 5-year) outcomes will be especially relevant in light of the safety questions that motivated SAFE-PAD [23]. Patients who required bailout/provisional stenting were excluded from the principal analysis. These cases represent clinically relevant acute failures of balloon-only strategies, and their exclusion introduces a degree of selection bias; the reported outcomes therefore reflect a cohort in whom the index balloon strategy was technically successful. The exact number of excluded bailout cases was not consistently captured as a discrete registry variable and cannot be reported with confidence; this precludes a formal intention-to-treat sensitivity analysis. True real-world patency and complication rates—inclusive of acute procedural failures—would likely be modestly less favorable in both arms. Lesion-level characteristics—lesion length, TASC II class, total occlusion status, severe calcification, run-off vessel score, and reference vessel diameters—are reported in Table 2 and were included in the multivariable adjustment, allowing direct comparability with K-POP [12] and the Wu series [15]. The study spans a four-year window (January 2021–December 2024), during which DCB technology continued to evolve. Procedural technique at our center remained consistent throughout the study period: the same pre-dilatation protocol, a minimum inflation time of 3 min, and a dual antiplatelet regimen were applied from the first to the last enrolled patient. However, we cannot fully exclude the possibility that minor center-level refinements in device sizing or lesion preparation accumulated over time, and the specific DCB devices used were not tracked as a formal study variable, precluding subgroup analysis by device generation or brand. A calendar-year sensitivity analysis to formally test for temporal allocation drift was not performed, as year-stratified subgroup sizes were insufficient for meaningful comparison.

Adequately powered, randomized, multicenter trials comparing DCB and POBA strategies in isolated popliteal and SFA disease, with mandated lesion-level characterization, IVUS guidance, protocol-mandated 6- and 12-month surveillance imaging, and at least 3-year follow-up, will be needed to definitively settle the long-term clinical and safety advantage of DCB in this segment. Sex-stratified, CKD-stratified, and lesion-length-stratified subgroup analyses would be especially informative, as would a head-to-head comparison of DCB strategies with and without prior atherectomy in the popliteal segment.

## 5. Conclusions

In a single-center, real-world cohort of 401 patients treated for isolated popliteal and/or SFA disease, DCB and POBA both delivered a mean ABI improvement of +0.27 at 12 months. The arms diverged on clinical outcome: DCB more often produced full symptomatic recovery (Rutherford 0 in 62.0% vs. 51.4%; *p* = 0.033) and roughly halved the odds of composite patency loss at 12 months (6.7% vs. 12.6%; unadjusted OR 0.50, 95% CI 0.25–1.01; chi-square *p* = 0.050), with the magnitude preserved after multivariable adjustment (adjusted OR 0.51, 95% CI 0.24–1.05; *p* = 0.065). Across this real-world cohort, the most robust between-arm difference was asymptomatic recovery at 12 months (62.0% vs. 51.4%; *p* = 0.033), which remained statistically significant after multivariable adjustment. Composite patency loss was numerically halved in the DCB arm but did not reach conventional significance either unadjusted (*p* = 0.050) or adjusted (*p* = 0.065), and should be interpreted as hypothesis-generating. A properly powered randomized trial—with lesion-level characterization, mandated surveillance imaging, and at least three years of follow-up—is still needed to settle the question definitively.

## Figures and Tables

**Table 1:** Baseline demographic and comorbidity characteristics.

Variable	POBA (*n* = 222)	DCB (*n* = 179)	*p*-Value
Age, years, mean ± SD	71.0 ± 9.8	65.3 ± 10.5	**<0.001**
Male sex, *n* (%)	172 (77.4)	144 (80.4)	0.462
Hypertension, *n* (%)	138 (62.1)	104 (58.1)	0.412
Heart failure, *n* (%)	42 (18.9)	26 (14.5)	0.235
Chronic kidney disease, *n* (%)	64 (28.8)	32 (17.8)	**0.009**
Diabetes mellitus, *n* (%)	118 (53.1)	92 (51.3)	0.720
Coronary artery disease, *n* (%)	98 (44.1)	78 (43.5)	0.911
Smoker, *n* (%)	152 (68.4)	128 (71.5)	0.495

Continuous variables presented as mean ± SD; categorical variables as *n* (%). Bold *p*-values are statistically significant (*p* < 0.05). DCB, drug-coated balloon; POBA, plain old balloon angioplasty.

**Table 2:** Lesion and procedural characteristics.

Characteristic	POBA (*n* = 222)	DCB (*n* = 179)	*p*-Value
**Lesion characteristics**			
Distal 1/3 superficial femoral artery involvement, *n* (%)	108 (48.6)	91 (50.8)	0.737
Lesion length, mm, mean ± SD	82.7 ± 43.3	94.5 ± 48.2	**0.010**
Proximal reference vessel diameter, mm, mean ± SD	5.3 ± 0.7	5.3 ± 0.7	0.784
Distal reference vessel diameter, mm, mean ± SD	4.6 ± 0.6	4.7 ± 0.6	0.050
Total occlusion, *n* (%)	43 (19.4)	71 (39.7)	**<0.001**
Severe calcification, *n* (%)	55 (24.8)	40 (22.3)	0.652
Run-off vessels ≤ 1, *n* (%)	77 (34.7)	69 (38.5)	0.487
**Popliteal segment involvement (among popliteal-involved patients)**			
P1 involvement, *n*/N (%)	30/52 (57.7)	38/62 (61.3)	0.851
P2 involvement, *n*/N (%)	39/52 (75.0)	48/62 (77.4)	0.943
P3 involvement, *n*/N (%)	20/52 (38.5)	27/62 (43.5)	0.736
**TASC II lesion type, *n* (%)**			
A	56 (25.2)	27 (15.1)	—
B	123 (55.4)	77 (43.0)	—
C	28 (12.6)	54 (30.2)	—
D	15 (6.8)	21 (11.7)	—
TASC II overall distribution	—	—	**<0.001**
**Index lesion territory, *n* (%)**			
Superficial femoral artery only	113 (50.9)	86 (48.0)	0.617
Popliteal artery only	15 (6.8)	14 (7.8)	0.838
Combined SFA + popliteal	37 (16.7)	48 (26.8)	**0.018**
Other/multi-segment	57 (25.7)	31 (17.3)	0.057
Contiguous infrapopliteal angioplasty	139 (62.6)	93 (52.0)	0.038
Common femoral artery involvement	16 (7.2)	19 (10.6)	0.305
Iliac territory involvement	24 (10.8)	26 (14.5)	0.318
**Wiring approach, *n* (%)**			
Intraluminal	195 (87.8)	150 (83.8)	0.310
Subintimal	27 (12.2)	29 (16.2)	0.310
**Device and adjuncts**			
Balloon nominal diameter, mm, mean ± SD	5.1 ± 0.7	5.2 ± 0.7	0.102
Inflation time, min, mean ± SD	2.4 ± 0.5	3.1 ± 0.4	**<0.001**
Adjunctive directional/rotational atherectomy, *n* (%)	1 (0.5)	4 (2.2)	0.251
Provisional bailout stenting	Excluded	Excluded	—
**Procedural outcome**			
Procedural success, *n* (%)	220 (99.1)	178 (99.4)	1.000
Post-procedural ABI, mean ± SD	0.93 ± 0.13	0.95 ± 0.13	0.137

Continuous variables presented as mean ± SD; categorical variables as *n* (%). Bold *p*-values are statistically significant (*p* < 0.05). DCB, drug-coated balloon; POBA, plain old balloon angioplasty; SFA, superficial femoral artery; TASC, Trans-Atlantic Inter-Society Consensus.

**Table 3:** Hemodynamic and clinical outcomes (ABI and Rutherford classification).

Outcome	POBA (*n* = 222)	DCB (*n* = 179)	*p*-Value
**Ankle–brachial index, mean ± SD**			
Baseline	0.69 ± 0.13	0.69 ± 0.13	0.892
6 months	0.98 ± 0.11	0.96 ± 0.11	0.162
12 months	0.96 ± 0.10	0.96 ± 0.10	0.757
ΔABI, baseline → 12 months	+0.27 ± 0.08	+0.27 ± 0.08	0.860
Within-group ABI change, baseline vs. 12 months (paired *t*-test)	<0.001	<0.001	—
**Rutherford classification, median (IQR)**			
Baseline	3 (3–4)	3 (3–4)	0.493
12 months	0 (0–2)	0 (0–1)	**0.048**
≥1 Rutherford category improvement, *n* (%)	187 (84.2)	156 (87.2)	0.409
Asymptomatic at 12 months (Rutherford 0), *n* (%)	114 (51.4)	111 (62.0)	**0.033**

ABI presented as mean ± SD; Rutherford as median (IQR) or *n* (%). Bold *p*-values are statistically significant (*p* < 0.05). ABI, ankle–brachial index; DCB, drug-coated balloon; IQR, interquartile range; POBA, plain old balloon angioplasty; SD, standard deviation.

**Table 4:** Twelve-month binary outcomes and unadjusted effect estimates.

Endpoint at 12 Months	DCB Events/*n* (%)	POBA Events/*n* (%)	OR (95% CI)	*p*-Value
Asymptomatic (Rutherford 0)	111/179 (62.0)	114/222 (51.4)	1.55 (1.04–2.31)	**0.033**
Composite clinical success	155/179 (86.6)	183/222 (82.4)	1.38 (0.79–2.39)	0.255
ABI ≥ 0.10 increase from baseline	177/179 (98.9)	218/222 (98.2)	1.62 (0.29–8.97)	0.575
≥1 Rutherford category improvement	156/179 (87.2)	187/222 (84.2)	1.27 (0.72–2.24)	0.409
Composite patency loss	12/179 (6.7)	28/222 (12.6)	0.50 (0.25–1.01)	**0.050**
Documented clinically driven TLR	8/179 (4.5)	16/222 (7.2)	0.60 (0.25–1.44)	0.251

Bold *p*-values are statistically significant (*p* < 0.05). Effect estimates are unadjusted odds ratios from 2 × 2 contingency tables. CI, confidence interval; DCB, drug-coated balloon; OR, odds ratio; POBA, plain old balloon angioplasty; TLR, target lesion revascularization.

**Table 5:** Multivariable logistic regression for 12-month composite patency loss.

Variable	Adjusted OR (95% CI)	*p*-Value
DCB use (vs. POBA)	0.51 (0.24–1.05)	0.065
Baseline ABI (per 0.1 unit)	1.94 (1.29–2.93)	**0.002**
Baseline Rutherford ≥ 4 (vs. 2–3)	3.28 (1.24–8.62)	**0.016**
Lesion length (per 10 mm)	1.01 (0.94–1.09)	0.757
Total occlusion (vs. non-occlusive)	1.10 (0.49–2.47)	0.818

Multivariable logistic regression with composite patency loss at 12 months as the dependent variable. Covariates: treatment arm, baseline ABI (per 0.1 unit), baseline Rutherford ≥ 4 (binary), lesion length (per 10 mm), and total occlusion (binary). Bold *p*-values are statistically significant (*p* < 0.05). ABI, ankle–brachial index; CI, confidence interval; DCB, drug-coated balloon; OR, odds ratio.

## Data Availability

The data presented in this study are available on request from the corresponding author. The data are not publicly available due to privacy and ethical restrictions.

## Abbreviations

The following abbreviations are used in this manuscript:

ABI	Ankle–brachial index
ANOVA	Analysis of variance
CFA	Common femoral artery
CI	Confidence interval
CIRSE	Cardiovascular and Interventional Radiological Society of Europe
CKD	Chronic kidney disease
CLTI	Chronic limb-threatening ischemia
CTA	Computed tomography angiography
CTO	Chronic total occlusion
DCB	Drug-coated balloon
DM	Diabetes mellitus
DUS	Duplex ultrasonography
EVT	Endovascular therapy
FP	Femoropopliteal
HF	Heart failure
HT	Hypertension
IQR	Interquartile range
IVUS	Intravascular ultrasound
OR	Odds ratio
PAD	Peripheral artery disease
POBA	Plain old balloon angioplasty
P1, P2, P3	Popliteal artery anatomical segments
RM-ANOVA	Repeated-measures analysis of variance
SD	Standard deviation
SFA	Superficial femoral artery
SIR	Society of Interventional Radiology
TASC	Trans-Atlantic Inter-Society Consensus
TLR	Target lesion revascularization
t-PA	Tissue plasminogen activator
